# Involvement of the renin-angiotensin system in the progression of severe hand-foot-and-mouth disease

**DOI:** 10.1371/journal.pone.0197861

**Published:** 2018-05-23

**Authors:** Chao Zhang, Shuaiyin Chen, Guangyuan Zhou, Yuefei Jin, Rongguang Zhang, Haiyan Yang, Yuanlin Xi, Jingchao Ren, Guangcai Duan

**Affiliations:** 1 Department of Epidemiology, College of Public Health, Zhengzhou University, Zhengzhou, Henan Province, China; 2 Department of Epidemiology, School of Public Health, Xinxiang Medical University, Henan Province, Xinxiang, China; Kliniken der Stadt Köln gGmbH, GERMANY

## Abstract

**Background:**

Hand-foot-and-mouth disease (HFMD) is generally considered as a mild exanthematous disease to infants and young children worldwide. HFMD cases are usually mild and self-limiting but for few cases leads to complicated severe clinical outcomes, and even death. Previous studies have indicated that serum Ang II levels in patients with H7N9 infection were related to the severity of infection. However, the mechanisms underlying the pathogenesis of severe HFMD remain unclear. This study was undertaken to clarify the role of the renin-angiotensin system (RAS) in the progression of severe HFMD.

**Methods:**

In the present study, 162 children including HFMD patients and healthy controls were recruited. The data was analyzed by time-series fashion. Concentrations of angiotensin II (Ang II) and noradrenaline (NA) in serum of patients were measured with ELISA. We established a mouse model for enterovirus 71 (EV71) infection and determined concentrations of Ang II, NA in tissue lysates at 3, 5 and 7 days post infection (dpi).

**Results:**

The concentrations of Ang II and NA in serum of the HFMD patients with mild or severe symptoms were significantly higher than that in healthy controls. Additionally, the concentrations of Ang II and NA in serum of severe cases were significantly higher than those mild cases and the increased concentrations of Ang II and NA showed the same time trend during the progression of HFMD in the severe cases. Furthermore, the concentrations of Ang II and NA in target organs of EV71-infected mice including brains, skeletal muscle, and lungs were increased with the progression of EV71 infection in mice. Histopathological alterations were observed in the brains, skeletal muscle and lungs of EV71-infected mice.

**Conclusion:**

Our study suggested that activation of the RAS is implicated in the pathogenesis of severe HFMD.

## Introduction

In recent years, several Hand-foot-and-mouth disease (HFMD) outbreaks have occurred in Asia-Pacific region and Europe [1–4]. Among them, enterovirus 71 (EV71) and coxsackie A16 (CA16) infection is main cause of HFMD outbreaks, and patients with EV71 infection are inclined to develop into severe symptoms [5, 6]. HFMD is generally considered as a mild exanthematous disease. Generally, most of HFMD cases are usually mild and self-limiting but for few cases viral infection leads to complicated clinical outcomes including brainstem encephalitis, aseptic meningitis, encephalitis, and acute flaccid paralysis (AFP), and even fatal cardiopulmonary failure[7–9].Numerous studies have shed light on the etiology and epidemiology of HFMD, and have helped medical professionals and public health officials worldwide understand this condition. Previous studies have found that EV71 enters into the digestive tract first and then enters the lymphatic system, and ultimately into the central nervous system (CNS)[10, 11]. CNS injury can lead to a rapid increase in intracranial pressure, increased sympathetic tone, and excessive activation of local renin-angiotensin system (RAS), further results in over-secretion of many cytokines into circulation, leading to peripheral vasoconstriction. Peripheral vasoconstriction will cause vascular endothelium injury and increased permeability, further inducing blood accumulation in the lower pulmonary region [12–15].

The RAS plays a critical action regulating the circulation in the human body in response to low blood pressure or decrease in serum sodium levels [16].A component of this system is angiotensinogen (AGT) that is synthesized and released by the liver into the general circulation. AGT is converted through another reaction into angiotensin I (Ang I) by the protein renin from the renal juxtaglomerular apparatus. Subsequently, Ang I is converted to angiotensin II (Ang II) by the pulmonary angiotensin-converting enzyme (ACE). AngII is an active octapeptide that acts primarily on Ang II receptor type 1 (AT1R) [16]. Angiotensin II (Ang II) is a key factor to activate AT1R, further inducing water and sodium storage by promoting the release of aldosterone and excessive activation of other neurohormonal system components by promoting the secretion of endothelin and noradrenaline (NA)[17, 18]. High concentration of NA in circulation is thought to associate with pulmonary edema [19], which may involve with fatal pulmonary edema in severe HFMD. The RAS can also activate related cells and regulates the expression of many mediators concerning cell growth and inflammatory responses [20, 21]. However, the role of RAS in the process of HFMD has not been well characterized yet. In the present study, we assumed that RAS participated in the progression of HFMD and tried to uncover the RAS-related potential mechanism in the progression of severe HFMD.

## Materials and methods

### Ethical statement

The study was reviewed and approved by the Life Sciences and Ethics Committee of Zhengzhou University and the Ethics Committee of the Zhengzhou Children’s Hospital. Written informed consent was obtained from each case’s guardian before enrollment.

### Study design

Total 162 subjects (10–60 months age) including 132 HFMD cases with mild (n = 79) or severe symptoms (n = 53) and healthy controls (n = 30) were recruited from Zhengzhou Children’s Hospital during April 2013 through June 2013. All HFMD cases were divided into different subsets of 1, 2, 3, 4, 5 days post infection (dpi) based on the hospitalization and initial onset time.

### Inclusion criteria

According to the “diagnosis and treatment guideline on hand-foot-and-mouth disease (2010)”, patients younger than 60 months with severe symptoms including meningitis, pulmonary edema, and mild cases without any nervous system lesions or pulmonary edema were included in this study. The children without any disease were classified as control.

### Exclusion criteria

The patients with congenital disease, acute or chronic hepatitis, cardiovascular disease, intestinal diseases, and other infectious diseases were excluded from this study.

### Animal model

BALB/c mice (SPF degree) were purchased from the Medical Animal Center in Zhengzhou University, Henan, China, and raised in individual ventilation cage (IVC) system. As described in our previous study[14, 15], 3-day-old BALB/c mice were i.p. inoculated with EV71 strain (2×10^6^ pfu/mouse) and sacrificed with isoflurane anesthesia on 3, 5 and 7 days post infection (dpi). The 3-day-old mice injected with the same volume of RD cell culture supernatants were used as controls and sacrificed with isoflurane on 3, 5 and 7 dpi.

### Tissue lysates

The brains, skeletal muscle and lungs of mice were ground into tissue homogenate with cold phosphate-buffered saline (PBS) at 4 ℃. Tissues were quickly removed and stored at -80 ℃. Tissues were grounded at 4 ℃ and repeated freezing and thawing for three times, followed by centrifugation at 3,000×g for 10 min at 4°C.

### Histopathological analysis

Mice were sacrificed on 3, 5, 7 dpi, and the brains, skeletal muscle and lungs of mice were immediately fixed in 4% paraformaldehyde at 4°C overnight. After fixation, paraffin-embedded tissues of 5 μm in thickness were stained with H&E.

### Measurement of Ang II and NA

The concentrations of Ang II and NA in serum or tissue lysates were measured by enzyme-linked immunosorbent assay (ELISA) kits (TSZ, Boston, USA).

### Data analysis

SPSS 17.0 (IBM, NC, USA) was used for the statistical analysis. One-way ANOVAs or Student’s t test depending on the validity of the normality assumption and the homogeneity of variance and a Pearson correlation analysis were performed. A significance level <0.05 was used for this study.

## Results

### General characteristics of the participants

One hundred thirty-two patients with HFMD were identified, including 79 mild HFMD patients, 53 severe HFMD patients, and 30 healthy children were used as controls. There was no significant difference in sex ratio (male ratio, controls: 1.31/1; mild: 1.47/1; severe: 1.35/1, *P*>0.05) and age (mouths, controls: 24.11±8.62; mild: 22.45±10.71; severe: 26.18±10.53, *P*>0.05) among these three groups.

### The concentrations of Ang II and NA were increased in serum of severe cases

**Fig 1:**
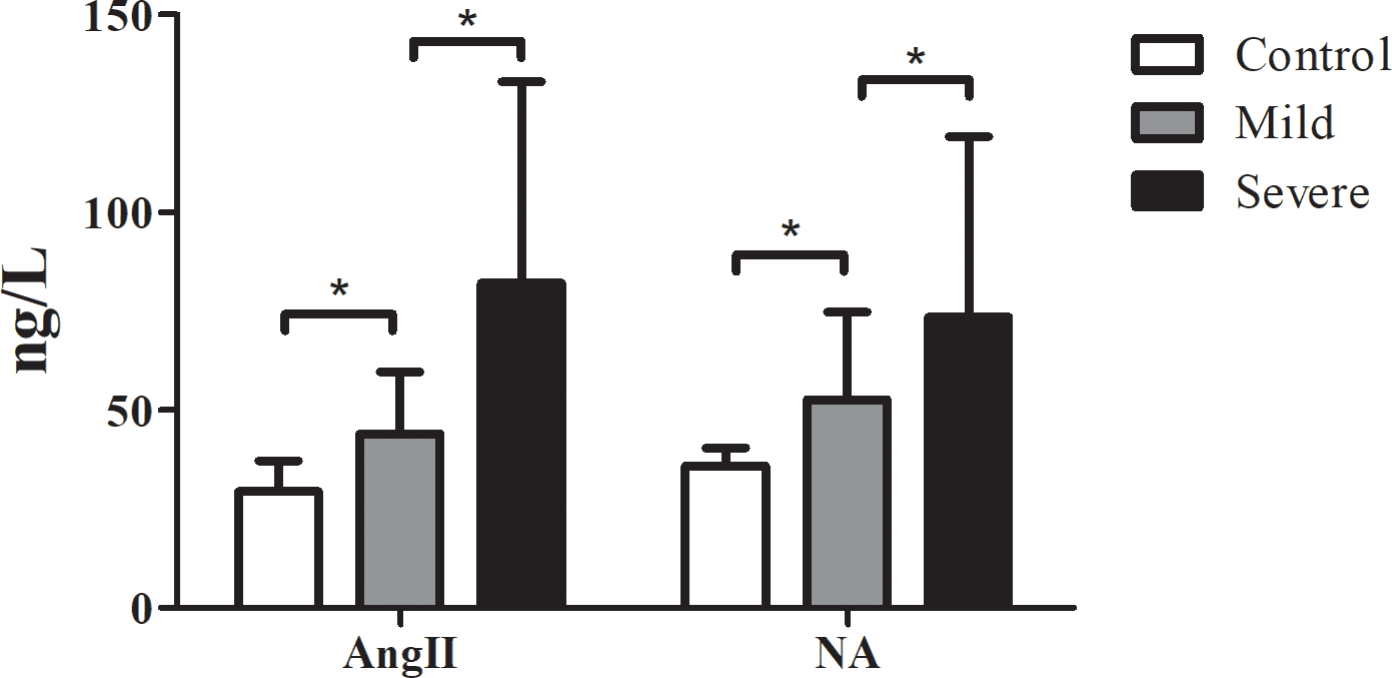
The concentrations of Ang II and NA in serum. The concentrations of Ang II and NA in serum were determined using ELISA kits. Data are expressed as mean ± SEM. *P<0.001.

**Fig 2:**
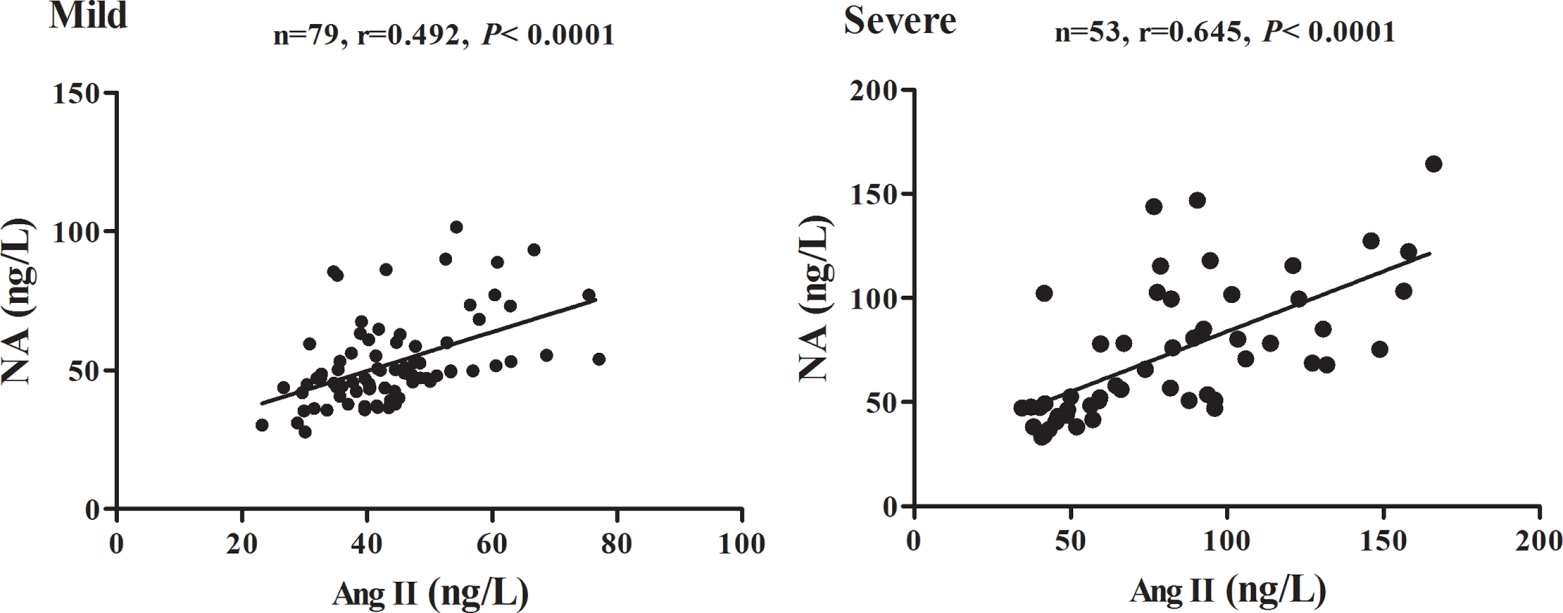
Correlation analysis of Ang II and NA. Pearson’s correlation test was used to analyze the relationship between Ang II and NA in serum of all subjects, r = Pearson correlation coefficient.

The concentrations of Ang II and NA are presented in Fig 1. The concentrations of Ang II and NA in serum of the HFMD patients with mild or severe symptoms were significantly higher than that in healthy controls (*P*<0.001). Additionally, the concentrations of Ang II and NA in serum of severe cases were significantly higher than those mild cases, *P*<0.05. Pearson correlation analysis (Fig 2) indicated that the concentration of Ang II was positively correlated to the concentration of NA during the progression of HFMD in both mild and severe (r = 0.492, *P*<0.001 for the mild; r = 0.645, *P*<0.001 for the severe). Together, our results suggested that the concentrations of Ang II and NA in serum were increased in HFMD cases.

### Time-series analysis of the concentrations of Ang II and NA in serum of HFMD

**Fig 3:**
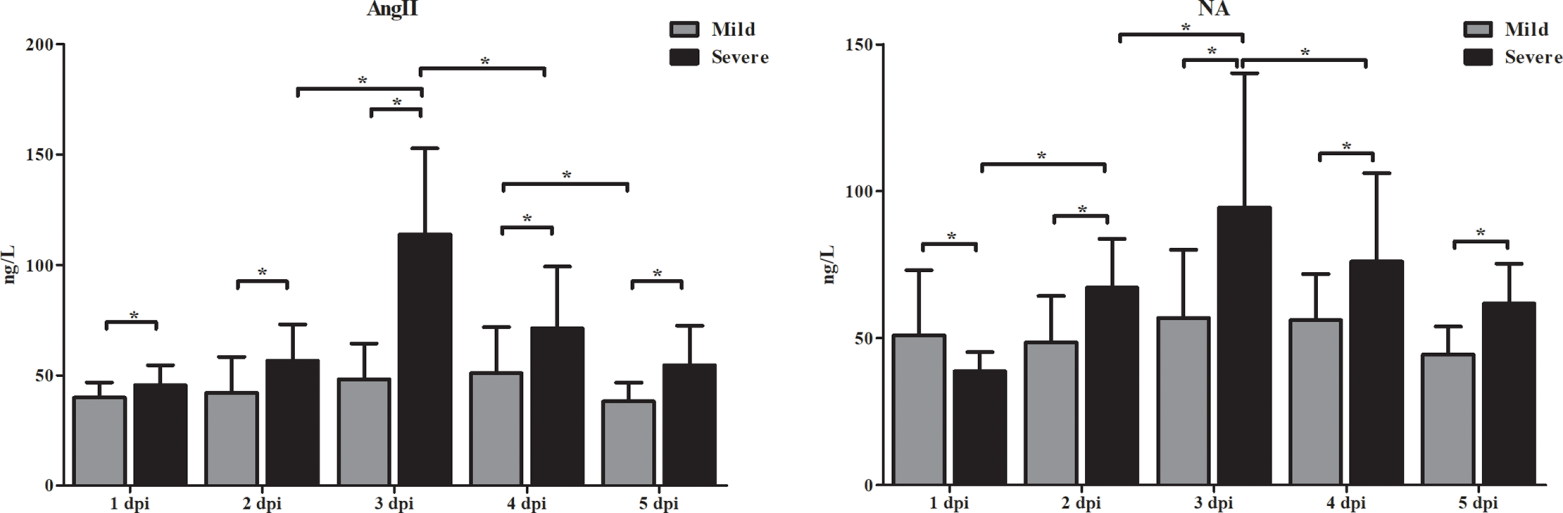
The concentrations of Ang II and NA in serum during the progression of HFMD. All HFMD cases were divided into different subsets of 1, 2, 3, 4, 5 days post infection (dpi) based on the hospitalization and initial onset time. The concentrations of Ang II and NA in serum of cases were determined using ELISA kits. Data are expressed as mean ± SEM. *P<0.05.

As shown in Fig 3, the concentration of Ang II in serum of the severe cases was significantly higher than that in the mild cases from 1 dpi to 5dpiduring the progression of HFMD. The concentrations of Ang II among the 5 subgroups of the severe were significantly different and increased from the 2 dpi to 4 dpi (*P*<0.001). The highest level of Ang II in serum of the severe cases occurred at 3 dpi. The concentrations of Ang II among the 5 subgroups in mild cases were not significantly different. The concentration of NA in serum of severe cases was significantly higher than those in mild cases during the progression of HFMD from 1 dpi to 5 dpi. The concentrations of NA among the 5 subgroups in severe cases were significantly different and increased from 1 dpi to 4 dpi (*P*<0.001). The highest level of NA in severe cases occurred at 3 dpi. The concentration of NA among the 5 subgroups in mild cases was no significant difference. Our results implied that increasing concentrations of Ang II and NA might participate in the progression of severe HFMD.

### The concentrations of Ang II and NA tissue lysates of EV71-infected mice

**Fig 4:**
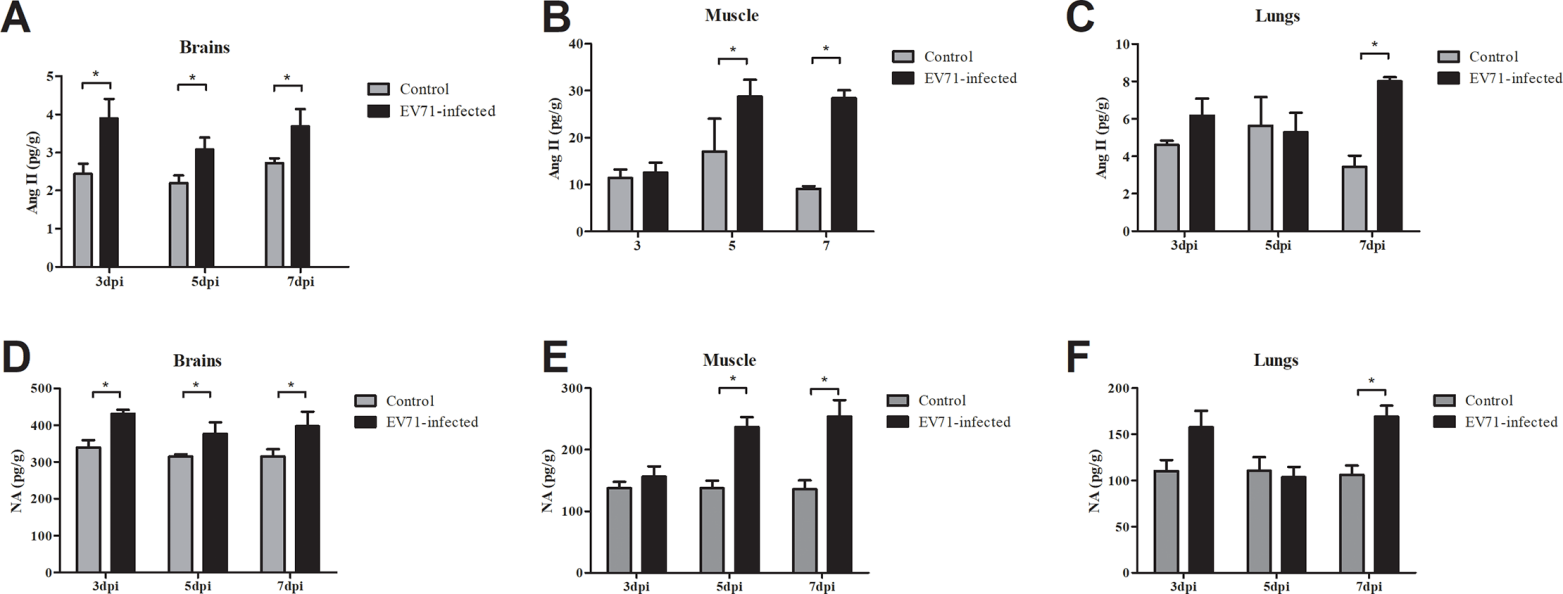
The concentrations of Ang II and NA in tissue lysates of EV71-infected mice. Mice were sacrificed on 3, 5, 7 dpi, and the brains, skeletal muscle and lungs of mice were removed. Then the tissue was weighted and lysated by cold PBS, followed by centrifugation at 3,000×g for 10 min at 4°C. The concentrations of Ang II and NA were determined using ELISA kits. Data are expressed as mean ± SEM. *P<0.05.

As shown in Fig 4, expression of Ang II and NA presented similar temporal trends in the corresponding tissues of EV71-infected mice. The concentrations of Ang II and NA in EV71-infected mice were significant increased in brains from 3 dpi to 7 dpi, skeletal muscle at 5 dpi and 7 dpi, lungs at 7 dpi compared to controls. The results demonstrated that EV71 infection increased Ang II and NA levels in target organs.

### Histopathological examination

**Fig 5:**
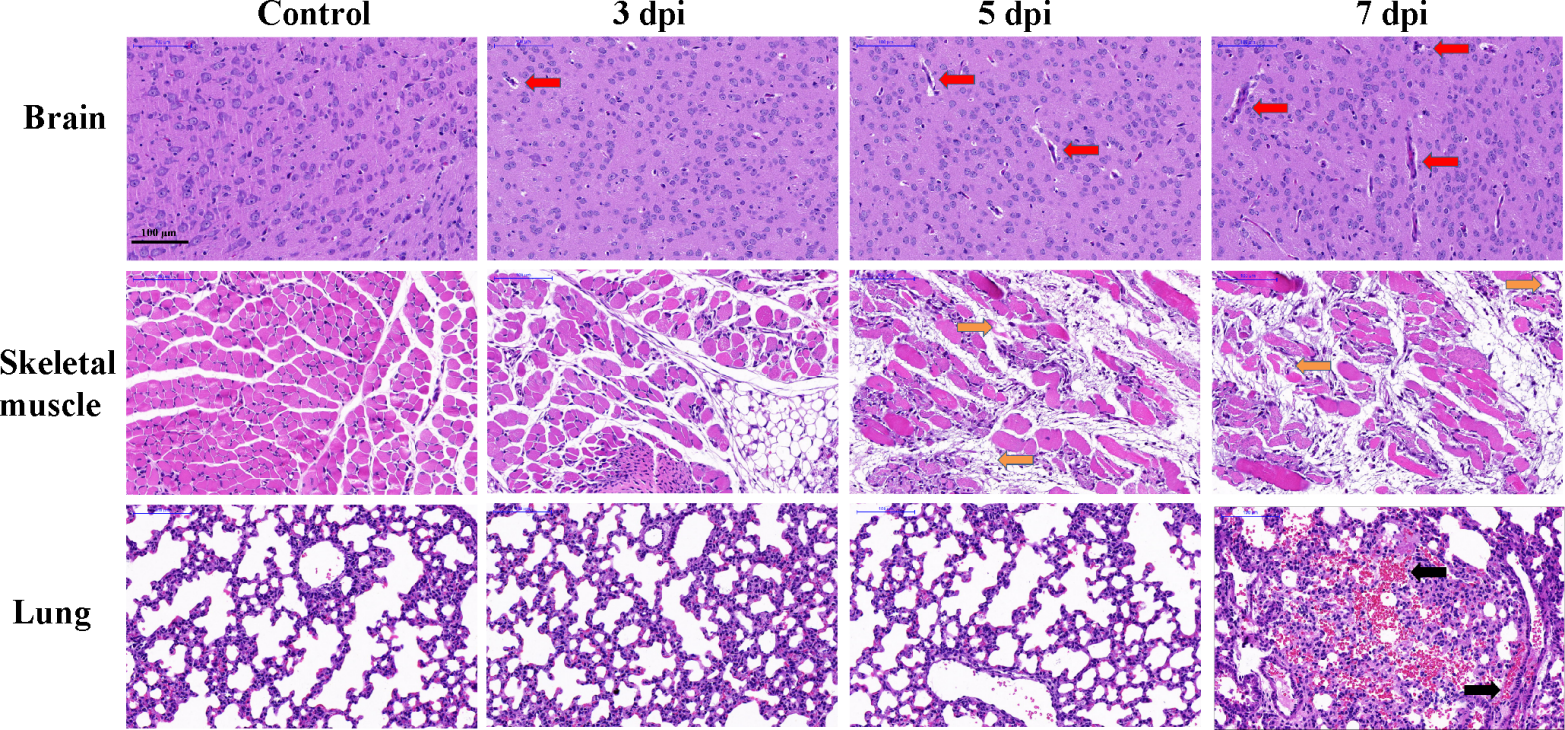
Histopathological alterations in brains, skeletal muscle, lungs of EV71-infected mice. Mice were sacrificed on 3, 5, 7 dpi. The removed organ or tissue were sliced and stained with H&E. Brain from EV71-infected mice exhibited perivascular cuffing (indicated by red solid arrows). Skeletal muscle from EV71-infected mice appeared necrotizing myositis with muscle fibers rupture (indicated by orange solid arrows) at 5 dpi and 7 dpi. Erythrocyte-filled fluid in the alveolar spaces was found in lungs (indicated by black solid arrows) from EV71-infected mice at 7 dpi. Bar = 100μm.

Histopathological alterations of mice at 3, 5, 7 dpi were examined with haematoxylin and eosin (H&E) staining. As shown in Fig 5, the brain tissues from EV71-infected mice exhibited pathological changes including perivascular cuffing compared with controls at 3, 5 and 7 dpi. Skeletal muscle from EV71-infected mice demonstrated necrotizing myositis with muscle fibers rupture and inflammatory cells infiltration at 5 dpi and 7 dpi. Lung lesions such as swollen alveoli and erythrocyte-filled fluid in the alveolar spaces were detected in lungs of EV71- infected mice at 7 dpi. The above results indicated that EV71 infection induced obvious histopathological alterations in target organs.

## Discussion

HFMD is a common viral infectious disease mainly caused by enterovirus 71(EV71) and coxsackievirus (CV) A16, which is mostly prevalent in infants and young children[9]. From 2008 to 2012, the mortality of severe HFMD is 1.9%, which is extremely higher than total mortality of HFMD [6, 22]. To date, no effective treatment for severe cases has been found. Thus, to explore early biomarkers to identify cases with potential to develop into severe symptoms will be beneficial to direct early clinical intervention and reduce mortality of HFMD. In the present study, we found that the concentrations of Ang II and NA were increased in serum of HFMD cases with mild or severe symptoms. Increasing concentrations of Ang II and NA in tissue lysates of mice were also determined during EV71 infection.

The current understanding of RAS is far more complex than its classic point, and two significant concepts have been added. First, the system is not only expressed at a systemic level but also works locally in a paracrine function in the vasculature, kidney, heart, lungs and CNS et al.[16]. In our study, we found that serum concentrations of Ang II and NA were elevated in HFMD cases, especially in severe cases, suggesting RAS activation. The concentration of Ang II was positively related to the concentration of NA during the progression of HFMD in both the mild and severe cases. The highest levels of Ang II and NA occurred at 3 dpi in HFMD cases. As shown in our previous study, the highest levels of some inflammatory cytokines occurred at 3 dpi in HFMD cases. In terms of above results, RAS activation may induce large amounts of pro-inflammatory cytokines [23]. Numerous studies on the pathogenesis of other viral diseases have also shown that pathogens can affect the stability of the RAS [24–27]. Thus evidence support our results, and activation of RAS may involve with the development of HFMD.

To further confirm the involvement of RAS in the pathogenesis of HFMD, we establish an EV71-infected mouse model [14, 15]. In our study, we found that EV71 infection induced Ang II and NA expression in the brains, skeletal muscle, and lungs of EV71-infected mice. We also observed histopathological alterations in these target organs, implying RAS activation may participate in the process of EV71 infection. Furthermore, pulmonary edema was also determined in EV71-infected mice. Previous studies have indicated that serum Ang II levels in patients withH7N9 infection were higher than controls and were related to the severity of infection [24, 26]. The S protein of SARS-CoV virus can down regulate the expression of ACE2 in the lungs and cause an accumulation of Ang II and acute lung injury (ALI). After the intervention of the ACE2 protein, symptoms of ALI were alleviated [25]. Zou, Z et al. found that with H5N1 infection, the RAS was involved and final concentration of Ang II were able to be used as prognostic indicators[27].These studies suggested that with viral infection, the pathogens could damage the homeostasis of the lung RAS, alter the local blood pressure and vascular permeability, and aggravate the imbalance of pro-inflammatory and anti-inflammatory mediators, thus affecting the occurrence and development of lung injury. Yumiko I et al. found that an excessive expression of Ang II can enhance the permeability of the lung through AT1R, resulting in pulmonary edema[28]. Together, above evidence suggest that EV71 infection-induced pulmonary edema may be an outcome of RAS activation.

## Conclusion

In summary, our study for the first time finds that activation of the RAS may involve in the progression of severe HFMD.
